# Immune biomarkers for epilepsy in autism: indications of cytokine alterations in an exploratory cross-sectional pediatric study

**DOI:** 10.3389/fneur.2025.1720712

**Published:** 2026-01-05

**Authors:** Marie K. Taylor, Filip Fredlund, Miriam Richter, Jenny Wickham, Olof Rask, Christine T. Ekdahl

**Affiliations:** 1 Division of Clinical Neurophysiology, Lund University, Lund, Sweden; 2 Department of Clinical Sciences Lund, Epilepsy Center, Lund University, Lund, Sweden; 3 Clinical Neurophysiology, Department of Medical Imaging and Physiology, Skåne University Hospital, Lund, Sweden; 4 Division of Child and Adolescent Psychiatry, Department of Clinical Sciences Lund, Lund University, Lund, Sweden

**Keywords:** autism spectrum disorder, biomarker, epilepsy, immune reaction, interleukin-13, interleukin-1, interleukin-12

## Abstract

**Background:**

Children with autism spectrum disorder (ASD) are at increased risk of epilepsy (EP), but distinguishing epileptic seizures from ASD-associated behavior remains a clinical challenge. Although previous studies have reported changes in peripheral immune markers in adults with EP, it remains unclear whether similar immune signatures are present in pediatric patients with both ASD and EP, and more pronounced than in children with ASD alone.

**Methods:**

We conducted an exploratory, prospective, cross-sectional study of children aged 9–14 years with mild ASD, with or without EP, recruited from outpatient settings. Peripheral blood samples were analyzed by enzyme-linked immunosorbent assay for 23 immune proteins and by flow cytometry for leukocyte population counts. Analyses included t-tests / Mann–Whitney U-tests, *post hoc* tests for multiple comparisons, and effect size / power analyses.

**Results:**

A total of 30 children were included, *n* = 21 with primarily mild ASD and *n* = 9 with mild ASD and EP. The epilepsy cases consisted of children with generalized seizures or self-limited epilepsy with centrotemporal spikes. Three immune proteins, Interleukin (IL)-12p70, IL-13 and IL-1β, were significantly increased in the ASD + EP group compared to the ASD-only group. However, the statistical power was low, and group differences did not remain significant after correction for multiple comparisons, even though effect sizes were moderate to large. No differences in the counts of activated leukocyte populations were observed.

**Conclusion:**

These findings raise the possibility that immune system alterations may be associated with EP in children with ASD and could potentially aid diagnosis, although larger studies are needed to confirm these findings.

## 1 Introduction

Epilepsy (EP) affects approximately 0.8% of the general population and 0.6% of children (1, 2). Among children with autism spectrum disorder (ASD), prevalence is markedly higher, with an estimated range of 5–46%, and the rate rises further in adults with ASD (3–6). ASD is a neurodevelopmental condition characterized by mild to severe difficulties in social interaction, repetitive behaviors and restricted interests (7).

Growing evidence implicates immune dysregulation both in disease pathogenesis and as a source of biomarkers for neurological disorders, including those of the central nervous system (8, 9). Circulating immune proteins in peripheral blood and cerebrospinal fluid, including cytokines and chemokines, are of particular interest. Members of the cytokine families, such as interleukin (IL)-6 /IL-12, guide T- and B-cell differentiation, whereas chemokines can recruit leukocytes across the blood–brain barrier to sites of neuroinflammation (8, 10, 11).

Previous studies have reported elevated blood levels of cytokines such as IL-6, IL-1β, IL12p70, IL-8 and IL-17 in children with ASD compared to typically developing controls (12–14). The role of inflammation in ASD is complex and may be influenced by comorbid conditions in which immune mechanisms are also implicated. Approximately 50% of children with ASD have comorbid attention deficit hyperactivity disorder (ADHD), and immune protein alterations have been observed both in ASD with ADHD, and in ADHD alone (15–17). Children with ASD also face an increased risk of psychiatric disorders such as depression and anxiety, which have likewise been linked to neuroinflammatory processes (18–20).

EP has similarly been investigated from a neuroimmunological perspective, with immune factors proposed as biomarkers (21–23). Both pro- and anti-inflammatory proteins are thought to play a role in epileptogenesis by modulating the excitatory/inhibitory balance in the central nervous system, potentially facilitating abnormal neuronal activity (24). In adults with EP, interictal peripheral blood studies have reported elevated levels of IL-6, IFN-*γ*, IL-17A, and TNF-*α* compared to healthy controls (25–28). IL-1β, among other cytokines, has been suggested as both an interictal marker and a potential therapeutic target in EP (29–31). However, findings remain inconsistent, with some studies reporting no significant changes or even decreased levels of cytokines such as IL-6, TNF-*α*, and IL-1β (27, 29, 32). Although pediatric studies are limited, altered cytokine levels, including TNF-α, IL-1β, and IL-1R1, have been reported in children with EP (32, 33).

The search for biomarkers to support the early diagnosis of epilepsy has gained momentum, particularly in light of diagnostic delays (34). This challenge is especially pronounced in children with ASD, where hospital/unfamiliar environments, clinical assessments and multiple electroencephalographies (EEGs) can be distressing. Moreover, distinguishing epilepsy symptoms from ASD-related behaviors further complicates diagnosis, underscoring the need for improved clinical tools.

It remains unclear, whether children with both ASD and EP exhibit a distinct immune profile in the blood compared to those with ASD alone. In this exploratory study, we examined immune protein levels and leukocyte activation in peripheral blood from children aged 9–14 years with mild ASD, with and without EP, to investigate potential biomarkers for EP in this population.

## 2 Method and materials

### 2.1 Patient inclusion

Patients were recruited between September 2020 and June 2023 through the Department of Pediatric Neurology at Skåne University Hospital (SUS) and Child and Adolescent Psychiatry in Skåne, Sweden. Inclusion criteria were: children aged 9–14 years with a confirmed diagnosis of ASD grade 1–2, with our without EP. Exclusion criteria were: severe developmental delay, traumatic brain injury within the last 6 months, active systemic autoimmune disease, or neurodegenerative disorders. Children with known monogenetic syndromes, other genetic disorders associated with ASD and/or EP, or treatment-resistant EP were not eligible for participation. Demographic data and medical history, including diagnoses, radiological findings, and perinatal data, were obtained from medical records. Child-adjusted study information was provided prior to enrolment. Written informed consent was obtained, and the study was approved by the Swedish Ethical Review Authority (approval no. 2020–03207) in accordance with the Helsinki declaration.

### 2.2 Blood sampling and biochemical analyses

Two blood samples were collected on one occasion per participant at the Department of Pediatric Neurology, SUS. Sample number 1 was collected in 5 mL BD Vacutainer serum tubes and were left to coagulate for 1 h at room temperature (RT) before centrifugation at 20 °C at 1500 rcf for 10 min. Serum was aliquoted and stored at −80 °C until analysis. Serum concentrations of cytokines and chemokines were determined by multiplex ELISA proinflammatory panel 1 (K15049D; INF-*γ*, IL1-*β*, IL-2, IL-4, IL-6, IL-8, IL-10, IL-12p70, IL-13 and TNF-*α*), chemokine panel 1 (K15047D; Eotaxin, Eotaxin-3, MIP-1α, MIP-1β, TARC, IP-10, MCP-1, MCP-4 and MDC) and vascular injury panel 2 (K15198D; SAA, CRP, VCAM-1 and ICAM-1) human kits according to the manufacturer’s instructions (MSD, US). Serum samples were diluted 1:2 (proinflammatory panel 1), 1:4 (chemokine panel 1) or 1:1000 (vascular injury panel 2) and run in duplicates along with calibrator standards. The samples were incubated at 4 °C overnight (proinflammatory panel 1, chemokine panel 1), or for 2 h in room temperature (vascular injury panel 2). After washing, detection antibody was added for 2 h at RT. The wash was then repeated and read buffer was added. Plates were read on the MESO QuickPlex SQ 120 and analyzed using discovery workbench 4.0 (MSD, US). Blood sample number 2 was collected in 5 mL whole blood EDTA (ethylenediaminetetraacetic acid tubes and stored at room temperature at a maximum of 24 h prior to flow cytometry analysis of circulating leukocytes). Gating was based on CD45 expression and side scatter (SSC) signature. Cell population counts included: B cells, CD4/CD8 ratio, CD4 + T cells, CD8 + T cells, HLA-DR + cells, HLA-DR + T cells, HLA-DR+/CD4 + T cells, HLA-DR+/CD8 + T cells, lymphocytes, and total T cells. The ELISAs were conducted in house at Lund Biomedical Center, while flow cytometry procedures were performed at the Department of Clinical Immunology and Transfusion Medicine, SUS.

### 2.3 Statistical analyses

Statistical analyses were conducted using statistical software R and SPSS Statistics version 27. Quantile-quantile plots and the Shapiro–Wilk test for normality were used to assess data distribution. For parametric distribution, data were analyzed with Student’s t-tests and presented as mean (SD), number of samples (*n*). For nonparametric/skewed distribution, including single outliers, data were analyzed with Mann–Whitney U-tests and presented as median (IQR), *n*. Undetectable values reported by the software were excluded from analysis. Cohen’s D was used for measuring effect sizes and Benjamini-Hochberg *post hoc* analysis for correction of multiple comparisons. Subgroup analyses were all performed with Mann–Whitney U-test due to few samples. *p*-values ≤0.05 were considered statistically significant.

## 3 Results

### 3.1 Patient inclusion and clinical characteristics

A total of 33 children were enrolled in the study. Following the exclusion of three participants due to diagnostic uncertainties or somatic comorbidities, the final cohort consisted of *n* = 30. Participants were divided into an ASD group (*n* = 21) and an ASD + EP group (*n* = 9). Clinical characteristics and medical history of the participants are presented in Table 1. A majority of participants were boys, namely 67% of ASD and 89% in ASD + EP groups, respectively. The majority of children in the ASD group and all in the ASD + EP group received additional support in school or attended adapted schooling. All participants were diagnosed with mild (grade 1) ASD, except for two in the ASD-only group who had moderate (grade 2) ASD. One child with ASD + EP had mild to moderate intellectual disability. Approximately two-thirds of participants in both groups had a comorbid diagnosis of ADHD or attention deficit disorder (ADD), and nearly half had received a psychiatric diagnosis such as anxiety disorder, obsessive-compulsive disorder and depressive disorder. Among the ASD + EP group, almost half had focal EP and the remainder had generalized EP. None of the included participants met the criteria for drug-resistant epilepsy (35). *N* = 6 reported seizure freedom of varying duration; none had seizures weekly at the time of enrollment. Other somatic diagnoses were few, and neuroimaging had been performed in only isolated cases.

**Table 1:** Clinical characteristics and medical history.

	Diagnosis	
	ASD (*n* = 21)	ASD + EP (*n* = 9)
**Clinical characteristics**		
Age: median years (range)	12 (9–14)	12 (9–14)
Gender: male % (*n*)	67 (14)	89 (8)
Schooling: % (*n*)		
Regular with no support	24 (5)	0 (0)
Regular with support	48 (10)	44 (4)
Adapted	14 (3)	56 (5)
Missing data	14 (3)	0 (0)
**Medical history**		
ASD level: % (*n*)		
Mild	90 (19)	100 (9)
Moderate	10 (2)	0 (0)
ADHD/ADD^a^: % (*n*)	71 (15)	67 (6)
Intellectual disability^b^: % (*n*)	0 (0)	11 (1)
Other psychiatric diagnosis^c^: % (*n*)	38 (8)	56 (5)
Prematurity^d^: % (*n*)	14 (3)	11 (1)
Missing data	14 (3)	22 (2)
Epilepsy classification: % (*n*)		
Focal		44 (4)
Generalized		56 (5)
MRT finding^e^: % (*n*)	0 (0)	33 (3)
Missing data	100 (21)	33 (3)
Somatic diagnosis^f^: % (*n*)	14 (3)	33 (3)
Medications^g^: median number (range)	2 (0–5)	3 (0–4)

Demography and medical history of included patients in the ASD and ASD+EP group. Data presented as median and range for age and medications, and as percentage (%) and number of participants (*n*) for all other parameters.

a *n* = 3 with attention deficit disorder (ADD) and *n* = 18 with attention deficit hyperactivity disorder (ADHD).

b *n* = 1 with moderate intellectual disability.

c *n* = 13, including *n* = 1 anxiety disorder, obsessive compulsive disorder and tics, *n* = 1 impressive and expressive language disorder, specific reading disorder and non-organic enuresis, *n* = 2 depressive disorder, *n* = 1 obsessive compulsive disorder, *n* = 1 depressive disorder and eating disorder, *n* = 1 pragmatic language disorder, *n* = 1 non-organic enuresis, *n* = 1 anxiety disorder, *n* = 1 non-organic sleeping disorder, *n* = 1 anxiety disorder and developmental coordination disorder, *n* = 1 mixed anxiety and depression conditions, and *n* = 1 generalized language disorder.

d *n* = 1 twin pregnancy born GW 35 + 6, *n* = 2 twin pregnancy born GW 36 + 4, and n = 1 born GW 33.

e *n* = 1 grey substance heterotopia, *n* = 1 cortical dysplasia, and *n* = 1 enlarged ventricles.

f *n* = 6 including *n* = 1 psoriasis, *n* = 1 unilateral hearing loss, *n* = 1 migraine, *n* = 1 lumbosacral dermal sinus, psoriasis and neuromuscular bladder disorder, *n* = 1 allergic rhinitis, and *n* = 1 bilateral sensorineural hearing loss.

g Medications in the ASD group included sertraline (*n* = 3), fluoxetine (*n* = 1), melatonin/circadin (*n* = 15), propiomazine (*n* = 1), metylphenidate (*n* = 6), guanfacine (*n* = 5), dexamphetamine (*n* = 4), atomoxetine (*n* = 3), hydroxyzine (*n* = 1), risperidone (*n* = 1), Grazax (*n* = 1), and over-the-counter allergy medications (*n* = 2). In the ASD+EP group, the following medications were reported: fluoxetine (*n* = 1), sertraline (*n* = 1), melatonin/circadin (*n* = 5), metylphenidate (*n* = 2), dexamphetamine (*n* = 3), guanfacine (*n* = 2), and risperidone (*n* = 1), and anti-seizure medication (ASM) (*n* = 5); lamotrigine (*n* = 2), levetiracetam (*n* = 1), oxcarbazepine (*n* = 2), valproic acid (*n* = 1). One ASD+EP participant was on ASM polytherapy with valproic acid and lamotrigine; the others were on monotherapy.

### 3.2 Indication of altered cytokine profile in children with ASD and EP compared to those with ASD only

**Figure 1:**
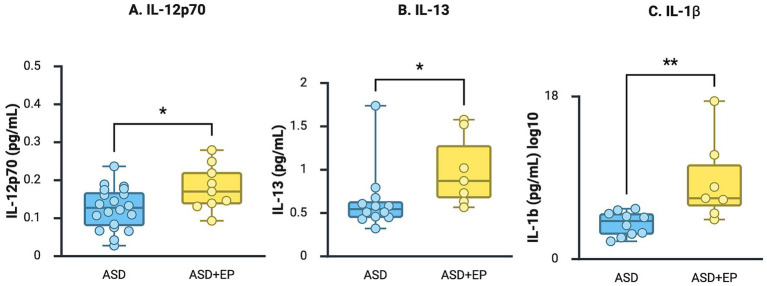
Immune proteins in serum. **(A–C)**. Scatter and box plots of IL-12p70, IL-13, and IL-1β serum concentrations in children with ASD and ASD + EP. Data presented with mean and SD for IL-12p70 due to normal distrubution and with median and IQR due to non-normal distribution for IL-13 and IL-1β. *p < 0.05, **p < 0.01. Figure was created in BioRender. Taylor, M. (2025) https://BioRender.com/cgu4kk8.

Analysis of 23 immune proteins in serum revealed significantly increased levels of three cytokines in the ASD + EP compared to the ASD-only group before correcting for multiple comparisons: IL-12p70 (*p* = 0.038, large effect size = 0.96, power = 0.6), IL-13 (*p* = 0.022, large effect size = 0.8, power = 0.33) and IL-1β (*p* = 0.005, medium-large effect size = 0.6, power = 0.22) (Figure 1). However, the differences did not remain statistically significant after correction for multiple comparisons (adjusted *p* = 0.29, 0.25, and 0.12, respectively). No other group differences were found, including IFN-*γ*, IL-6, IL-8, TNF-*α*, and MCP-1 (Table 2). The detected levels of immune proteins were broadly consistent with previous reports for some of these proteins in pediatric cohorts (36). No participants were excluded due to high C-reactive protein (CRP), which could have indicated ongoing systemic infection.

**Table 2:** Protein levels in serum.

Protein (mg/dl, pg/ml) (min–max)	ASD	ASD+EP	*p*-value
CRP* (mg/dl) (0.3E-6–1.2E6)	0.02 (1.64), 21	0.0008 (0.04), 9	0.33
Eotaxin (pg/ml) (119.25–510.72)	230.34 (66.79), 21	241.91 (120.89), 9	0.79
Eotaxin-3 (pg/ml) (6.97–33.83)	18.75 (6.3), 21	19.72 (7.37), 9	0.74
ICAM-1* (pg/ml) (0.66–427.94)	25.72 (52.72), 21	55.42 (180.14), 9	0.16
IFN-g (pg/ml) (3.26–65.77)	9.91 (13.95), 21	5.23 (1.93), 9	0.15
IL-10* (pg/ml) (0.07–0.90)	0.29 (0.16), 21	0.27 (0.18), 9	0.76
IL-12p70 (pg/ml) (0.03–0.28)	0.13 (0.05), 20	0.18 (0.06), 9	**0.04
IL-13* (pg/ml) (0.32–1.74)	0.55 (0.19), 12	0.87 (0.59), 7	**0.02
IL-1β* (pg/ml) (0.01–14.56)	0.05 (0.05), 10	0.16 (0.63), 7	**0.01
IL-2* (pg/ml) (0.06–1.33)	0.19 (0.09), 17	0.29 (0.18), 8	0.12
IL-4* (pg/ml) (0.0045–0.13)	0.03 (0.02), 17	0.02 (0.01), 9	0.22
IL-6* (pg/ml) (0.19–3.82)	0.63 (0.36), 21	0.48 (0.76), 9	0.79
IL-8 (pg/ml) (5.64–18.53)	9.53 (3.27), 21	10.12 (3.73), 9	0.69
IP-10 (pg/ml)* (57.90–757.00)	125.63 (59.90), 21	100.90 (43.54), 9	0.33
MCP-1* (pg/ml) (134.72–294.16)	208.67 (61.28), 21	217.90 (27.64), 9	0.37
MCP-4* (pg/ml) (24.12–182.45)	61.48 (28.92), 21	62.35 (20.08), 9	0.26
MDC (pg/ml) (811.23–2204.67)	1403.59 (323.60), 21	1209.11 (407.43), 9	0.23
MIP-1a* (pg/ml) (8.02–98.50)	18.45 (7.31), 20	19.47 (5.32), 9	0.44
MIP-1b (pg/ml) (71.17–256.18)	118.95 (41.39), 21	135.89 (43.61), 9	0.34
SAA* (mg/dl) (0.0014–2.67E8)	0.19 (8.05), 21	0.59 (2.91), 9	0.76
TARC (pg/ml) (81.16–1144.02)	409.87 (260.52), 21	331.26 (162.35), 9	0.33
TNF-a (pg/ml) (0.86–3.44)	2.26 (0.60), 21	2.51 (0.62), 9	0.34
VCAM-1* (pg/ml) (5.69–1856.26)	119.01 (147.39), 21	209.62 (1114.73), 9	0.11

Protein levels analyzed by ELISA in children with ASD and ASD+EP. Data presented as mean (SD), *n*. *Data presented as median (IQR), *n*. **p-value before adjusted for multiple comparisons.

Subgroup analyses were performed separately within the ASD + EP and ASD-only group, based on gender, comorbid of ADHD/ADD, psychiatric or somatic disorders, and focal versus generalized EP. The following proteins were selected based on previous findings identifying them as potential biomarkers: IFN-*γ*, IL-6, IL-12p70, IL-8, TNF-*α*, and MCP-1 (16, 25, 28, 29). However, apart from increased TNF-α levels in participants with ASD and comorbid ADHD compared to those without ADHD (2.26 ± 0.60, *p* = 0.036), no significant differences were found (data not shown). Subgroup analyses for IL-1β and IL-13 were not conducted due to low overall serum detection rates in both patient groups (*n* = 7–12).

### 3.3 No differences in the counts of activated leukocyte populations between children with ASD with or without EP

**Figure 2:**
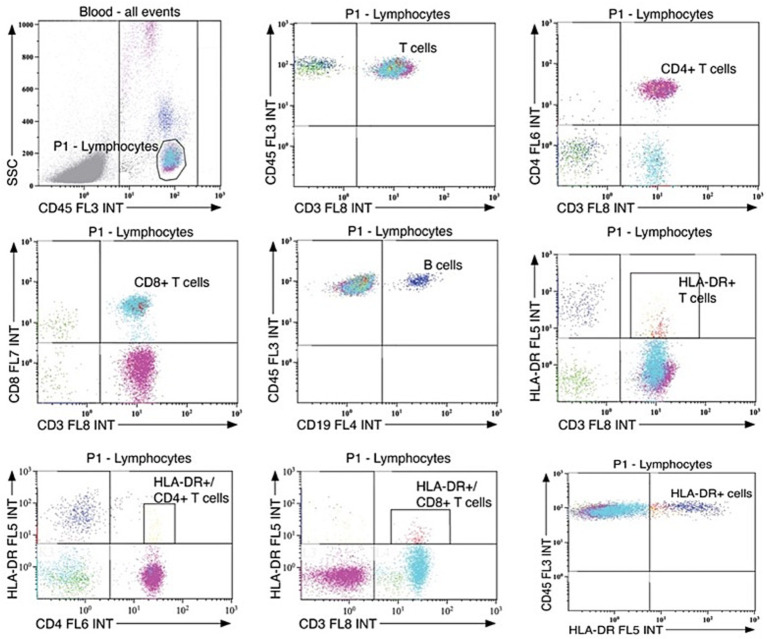
Gating strategies for leukocyte population counts. Standardized gating strategies for flow cytometry of leukocyte populations in serum.

The gating strategies used to isolate cell populations is shown in Figure 2. Leukocytes were gated based on their CD45 expression and side scatter (SSC) signature. The following subpopulations were analyzed: HLA-DR^+^ cells (CD45^+^HLA-DR^+^), B-cells (CD45^+^CD19^+^), T-cells (CD45^+^CD3^+^), CD4^+^ T-cells (CD3^+^CD4^+^), CD8^+^ T-cells (CD3^+^CD8^+^), and HLA-DR^+^ T-cell subsets (HLA-DR^+^CD3^+^, HLA-DR^+^CD4^+^, and HLA-DR^+^CD8^+^).

Flow cytometry analysis revealed no significant differences in circulating leukocyte levels between the ASD + EP and ASD-only group (Table 3). Total counts of HLA-DR^+^cells, HLA-DR^+^ T-cells, and subsets of HLA-DR^+^/CD4^+^ and HLA-DR^+^/CD8^+^ T-cells, showed substantial variability within both groups. The leukocyte counts in our cohort were in line with previously established pediatric reference values (37).

**Table 3:** Leukocyte population counts in serum.

Cell population (x 10^9^/L)	ASD	ASD+EP	*p*-value
B cells*	0.32 (0.27), 21	0.26 (0.21), 9	0.15
CD4/CD8 ratio	1.91 (0.43), 21	1.89 (0.50), 9	0.94
CD4 + T cells	1.00 (0.30), 21	0.86 (0.22), 9	0.18
CD8 + T cells	0.54 (0.17), 21	0.48 (0.18), 9	0.41
HLA-DR + cells*	0.39 (0.28), 21	0.31 (0.26), 9	0.15
HLA-DR + T cells*	0.04 (0.04), 21	0.05 (0.04), 9	0.66
HLA-DR+/CD4 + T cells*	0.02 (0.02), 21	0.02 (0.01), 9	0.76
HLA-DR+/CD8 + T cells*	0.01 (0.01), 18	0.02 (0.02), 9	0.32
Lymphocytes*	2.14 (1.34), 21	1.88 (0.61), 9	0.24
T cells	1.72 (0.50), 21	1.44 (0.36), 9	0.11

Cell populations analyzed by flow cytometry in children with ASD and ASD+EP. Counts are presented as cells × 10^9^/L unless otherwise stated. Data presented as mean (SD), *n*. *Data presented as median (IQR), *n*. CD, cluster of differentiation; HLA, human leukocyte antigen.

## 4 Discussion

In this study, we explored 23 immune parameters significant for neuro-inflammation in a cohort of 30 children aged 9–14 years with ASD with or without EP. All participants had mild ASD, except for two in the ASD-only group with moderate ASD. The ASD + EP group included cases of both focal and generalized EP. Of the 23 immune proteins analyzed, three cytokines IL-12p70, IL-1β and IL-13, were significantly elevated in the ASD + EP group compared to the ASD group, although differences did not remain significant after correction for multiple comparisons. No group differences were observed in leukocyte population counts. Thus, these findings indicate three candidate markers for EP in ASD which warrant further study in larger, confirmatory cohorts.

Previous studies have shown altered immune reactions and functioning in both ASD and EP. In addition, neuroinflammatory processes are of special importance in the context of pediatric EP (38). Given that participants with both ASD and EP were included in our study, a certain degree of overlap in immune activity could be expected. The observed increase in IL-12p70, IL-1β and IL-13 in ASD + EP children, before adjustment for multiple comparisons, suggest enhanced pro- and anti-inflammatory activity associated with EP. The results align with a recent study on adults with temporal lobe EP (39). No group differences were observed for the other evaluated cytokines and chemokines. IL-6, a cytokine frequently studied in the context of neuroinflammation, has previously been reported to be elevated in both ASD and EP (13, 40). In our study, the effect size for IL-6 was moderate (=0.54), suggesting that a chronic increase may have been present in both groups, potentially explaining the absence of significant group differences and thus limiting the relevance of IL-6 in this particular patient cohort. However, larger studies are needed to confirm this.

In subgroup analysis, the only significant association was between a co-diagnosis of ADHD and TNF-*α* levels within the ASD group. In a previous study of 13 children with ASD and 9 with ASD + ADHD, those with comorbid ADHD had increased levels of proinflammatory cytokine MIF and decreased levels of chemokine IL-8, while children with ASD only showed increased levels of MCP-1 compared to typically developing controls (16). Studies focusing on ADHD have also linked inflammatory proteins to certain biotypes, although how such patterns may interact with comorbid EP remains unknown (15).

Previous studies on the immune reactions in adults undergoing clinical evaluation and video-EEG recordings for frequent seizures have shown that immune profiles differ between interictal and postictal blood sampling (28, 29). Interictal cytokine levels have also be associated with seizure recurrence risk and severity, including IL-1β in children with EP (29, 41, 42). According to medical records and parental reports, all children with EP in the present study had adequate seizure control at the time of enrollment. However, no continuous video-EEG confirmed seizure activity or freedom on the day of blood sampling, which may have influenced immune marker levels. Transient increases in immune factors have been observed postictally after focal seizures (28), and adults with focal EP have shown transient postictal increase in immune cell counts (43). A retrospective study of adults also indicated higher leukocyte count in generalized versus focal EP (44). Due to small sample size in our study, comparisons between focal and generalized EP in the ASD + EP group had low statistical power and need further exploration. The neutrophil-to-lymphocyte ratio (NLR) has also been suggested as a potential biomarker for EP. While some studies have reported increases in NLR, results remain mixed, and the literature is heterogenous (45). In the present study, NLR could not be evaluated due to lack of neutrophil counts. Although technical factors cannot be entirely excluded as an explanation, the substantial variability in leukocyte population counts, is still in line with previously described normal data and variability (37) and may be explained by biological heterogeneity.

Most studies of immune responses in pediatric EP have focused on severe cases, such as epileptic seizures associated with autoimmune encephalitis or febrile infection-related epilepsy syndrome (46). By contrast, the children in our study had relatively mild forms of EP, such as self-limited epilepsy with centrotemporal spikes and generalized absence epilepsy. It is possible that the immune responses observed in this group were also influenced by their underlying ASD. Previous studies have reported elevated immune factors and changes in immune cell populations in children with ASD (12, 13), but these findings are primarily based on young children with more severe ASD diagnosis, potentially reflecting a different immunological profile. Altered immune profiles have also been reported in post-mortem brain tissue from individuals with moderate to severe ASD (47). In contrast, the present study focused on children with mild ASD, which may involve different immune mechanisms. Additionally, many previous studies did not provide information on comorbid EP, limiting direct comparisons.

This exploratory study has some limitations: First, the relatively small sample size limits statistical power, especially given the heterogeneity in prior research. Technical concerns such as low detection rates in certain samples including IL-1β and IL-13 warrants careful interpretation of the results. Second, the study had a skewed gender distribution, with few female participants, which may reduce the generalizability of the findings. Third, the absence of neutrophil counts prevented evaluation of the NLR. Fourth, a potential confounder in this study is the high prevalence of psychiatric medications and ASMs, both of which have previously been reported to affect levels of immune proteins (48, 49).

## 5 Conclusion

Although systemic immune proteins levels may have potential as diagnostic markers for EP, even in milder cases with infrequent seizures, the presence of neuropsychiatric disorders, such as ASD, complicates the interpretation. Given the exploratory nature of this study, further research is warranted to better understand the immune profile associated with EP in the context of ASD.

## Data Availability

The raw data supporting the conclusions of this article will be made available by the authors, without undue reservation.
